# Measuring performance in allied health professional role substitution models of care: a clinician survey

**DOI:** 10.1186/s12913-024-10556-5

**Published:** 2024-01-16

**Authors:** Rumbidzai N. Mutsekwa, Katrina L. Campbell, Russell Canavan, Rebecca L. Angus, Liza-Jane McBride, Joshua M. Byrnes

**Affiliations:** 1 Gold Coast Hospital and Health Service, Nutrition and Food Services, 1 Hospital Boulevard Southport, Southport, Queensland 4215 Australia; 2 Gold Coast Hospital and Health Service, Allied Health Research Team, 1 Hospital Boulevard Southport, Southport, Queensland 4215 Australia; 3 Centre for Applied Health Economics, School of Medicine, Sir Samuel Griffith Centre, Griffith University, Nathan, Queensland 4111 Australia; 4 Menzies Health Institute Queensland, Griffith University, Gold Coast Campus, Southport, Queensland 4215 Australia; 5 Metro North Hospital and Health Service, Healthcare Excellence and Innovation, 153 Campbell Street, Bowen Hills, Queensland 4029 Australia; 6 Gastroenterology Department, Gold Coast Hospital and Health Service, 1 Hospital Boulevard Southport, Southport, Queensland 4215 Australia; 7 School of Allied-health Sciences and Social Work, Griffith University, Gold Coast Campus, Southport, Queensland 4215 Australia; 8 Department of Health, Clinical Excellence 15 Butterfield Street, Herston, Queensland 4006 Australia

**Keywords:** Professional role substitution, Extended scope, Expanded scope, Evaluation, Measuring performance

## Abstract

**Background:**

Professional role substitution models of care have emerged as a key strategy to address increasing healthcare demand. Gaining insights from those actively engaged in the process of these models’ implementation and evaluation is pivotal to ensuring sustainability and further successful implementation. The purpose of this study was to describe allied-health clinicians’ perceptions, practice, and experiences of healthcare performance evaluation in professional role substitution models of care.

**Methods:**

Data were collected via an online platform between 22 June − 22 July 2022 using a combination of convenience and network-based sampling of allied-health clinicians involved or interested in the implementation and evaluation of professional role substitution models of care. Clinicians answered 25 questions which consisted of demographic and targeted questions regarding performance evaluation across six domains of healthcare quality (effectiveness, safety, appropriateness, access & equity, continuity of care, and cost, efficiency, productivity & sustainability).

**Results:**

A total of 102 clinicians accessed the survey, with 72 providing complete survey data. Eleven allied-health professions were represented, working across twelve specialities in thirteen hospital and health services. Whilst most allied-health clinicians (93–100%) supported measuring performance in each of the six healthcare quality domains, only 26–58% were measuring these domains in practice. Allied-health leadership support (62.5%), clinician drive (62.5%), consumer engagement (50%) and medical support (46%) were enablers whilst a lack of resources (human, time, financial (47%)), healthcare performance frameworks and/or policies (40%) were identified as barriers. Given the opportunity, clinicians would invest the most financial resources in digital solutions as a core strategy to improve performance evaluation.

**Conclusions:**

Allied-health professionals expressed strong support for principles of performance evaluation, however in practice, performance evaluation is still in its infancy in professional role substitution models of care. Organisations can implement strategies that maximise the enablers whilst addressing barriers identified to improve performance evaluation in these models of care.

**Supplementary Information:**

The online version contains supplementary material available at 10.1186/s12913-024-10556-5.

## Background

In contemporary healthcare systems professional role substitution has emerged as a pivotal paradigm shift, wherein non-medical healthcare professionals, such as physician assistants, nurses, and allied health practitioners, assume responsibilities traditionally reserved for medical doctors or specialist physicians [1]. This has become a key strategy to address the escalating demands on healthcare services and the impending scarcity of medical workforce [2, 3]. Furthermore, this shift is closely aligned with the principles of value-based healthcare (VBHC), a philosophy centred on the imperative to strengthen healthcare systems for sustainability while simultaneously delivering high-quality care [4–6]. The essence of quality healthcare lies in its ability to provide safe, timely, cost-effective care that demonstrably yields positive patient outcomes [4, 7, 8]. 

Over the past two decades, the deployment of allied health professional role substitution models has surged, enhancing patient access to care across diverse medical specialties and liberating medical specialists to focus on more complex or urgent cases. Furthermore, a growing body of evidence highlights the safety and effectiveness of these allied health models, and they are also well-received by patients [9–11]. Nonetheless, the literature supporting these models often falls short in terms of comprehensive performance measurement, lacking the robust evaluation required to gauge their impact effectively [9, 10]. A recent systematic review underscored the inadequacy of performance measurement in professional role substitution models, particularly concerning established constructs and domains of healthcare quality, such as effectiveness, safety, access, appropriateness, continuity, efficiency, sustainability, and cost of care [7]. 

For allied health professional role substitution models to gain credibility as viable, legitimate, and effective alternatives in healthcare delivery, it is imperative to demonstrate that the experiences of receiving and providing care do not deteriorate when compared to traditional medical models. Performance evaluation and transparent reporting are instrumental in enhancing implementation, delivery, adoption, sustainability and outcomes of these models [4]. At present, there is a glaring absence of agreed-upon approaches for measuring or monitoring the performance of allied health professional role substitution models in practice, necessitating a systematic and coordinated approach to ascertain their value in healthcare delivery settings. This endeavour is vital for accumulating the evidence needed to inform continued or future commissioning of these services [5, 7, 8, 12]. 

Beyond the traditional purview, there exists a compelling case for a broader perspective—one that acknowledges and validates measures of paramount importance to patients, clinicians, and other stakeholders, including healthcare managers, policy makers, and payers. Allied health professional role substitution models often not only meet but surpass patients’ expectations by delivering cost-effective care, timely access, effective treatments, reassurance, enhanced care experiences (e.g., time spent with clinicians, clinical expertise, professional demeanour, and interpersonal skills) [11, 13]. However, limited research has delved into allied health clinicians’ perceptions and experiences concerning performance evaluation aimed at demonstrating the quality of care delivered. Gaining insights from those actively engaged in these models’ implementation and evaluation processes is pivotal to ensuring further successful implementation and sustainability.

This study seeks to address these critical gaps by (i) elucidating allied health clinicians’ perceptions, practices, and experiences related to performance evaluation; (ii) identifying the enablers, and barriers that shape robust performance evaluation; and (iii) pinpointing and prioritising key strategies capable of bolstering performance evaluation within allied health professional role substitution models.

## Methods

### Study design and participants

An anonymous online survey was designed to include a combination of closed and open-ended questions. The target population for this study comprised allied health clinicians who were actively engaged or had an interest in the implementation, evaluation, and performance measurement of professional role substitution within the healthcare context of Queensland, Australia which has approximately 35 000 allied professionals [14]. In 2014, Queensland Health initiated an expanded scope of practice strategy, leading to the development of approximately 133 distinct care models as reported in the 2018–2019 annual activity data reports [1, 15, 16]. 

### Survey development and validation

To bolster the survey’s validity and reliability, a validation process was undertaken following the seven-step protocol outlined in the Association of Medical Education in Europe (AMEE) guide “Developing Questionnaires for Educational Research: AMEE Guide No. 87” [17]. This process encompassed the following steps:

1. A targeted systematic literature review was conducted to define survey concepts and constructs, as well as identify scales that could be adapted for use in the survey [7].
2. Interviews were conducted with potential respondents to ensure that survey constructs aligned with how participants would conceptualise and comprehend them.
3. Information synthesis was carried out based on the literature review findings and insights gathered from interviews.
4. Survey items were developed based on the synthesised information.
5. Expert validation was conducted using a combination of qualitative and quantitative methods from a panel of five experts in professional role substitution implementation and evaluation They provided feedback on whether the survey instrument appeared at “face value” to measure what it aimed to e.g., (relevance purpose, audience) and layout (face validity). They also assessed how adequately the survey captured the theoretical concepts and the extent to which they were multi-dimensional or multi-faceted (construct validity). Lastly experts assessed how fully the survey questions measured the constructs of interests and the key items had not been omitted (construct validity). Experts were asked to state whether each item was essential, useful but not essential or not necessary. The content validity ratios (CVR) were calculated for each proposed survey item where *CVR*=(*Ne*​−*N*/2)​/(N/2), and *Ne*​ is the number indicating an item as essential and *N* is the total number of experts. Twenty-five survey items met the minimum value of content validity ratio of 0.99 necessary from five expert panel members for statistical significance at p < 0.05 and were included in survey instrument [18].
6. The interpretation of survey items and response anchors was assessed with potential respondents through cognitive interviewing, using the “think out loud” technique, to validate the response process. Analysis was by coding and interpretation of notes from interviews.
7. Pilot testing was conducted with four prospective respondents at two different time points. This was done using the planned delivery mode; an online survey platform REDCap. A high correlation of 0.9 indicated a good test-retest reliability with only minor wording refinements required.

### Survey content

The survey (Additional file 1) included sociodemographic questions and addressed clinicians’ perceptions, practices, and experiences of performance evaluation in professional role substitution (including perceived barriers and enablers). Participants were asked to rank five potential strategies known to support performance evaluation, by distributing $100 of hypothetical funding across these. This was modelled on an Australian Bureau of Statistics question used to consider policy preferences. The 25-question survey consisted of multiple-choice, categorical and Likert scale options to reduce participant burden and risk of incomplete answers. Open ended questions were integrated to capture unanticipated responses [19]. 

### Ethical consideration

This study adhered to the principles outlined in the Declaration of Helsinki and received ethical approval from both the Gold Coast Hospital and Health Service (HREC/2020/QGC/62,104) and Griffith University (GU Ref No: 2020/876). The study also followed the Consensus-Based Checklist for Reporting of Survey Studies (CROSS) [20]. 

### Survey administration

Data collection was facilitated through the REDCap online survey management platform. A combination of convenience and network based sampling was adopted, with invitations and survey links distributed via email through various State, hospital, and professional networks, including Allied Health Professions Queensland (AHPOQ) and expanded scope of practice professional working groups. Respondents were also invited to share links within their networks Participants were provided with a study information sheet outlining the study’s objectives and procedures, emphasising the voluntary nature of participation. A reminder email was sent three weeks after the initial invitation. Participants were offered the opportunity to enter a draw for one of three AUD$100 gift vouchers. No personally identifiable information was collected and contact details of participants expressing interest in further research or the gift voucher draw were not linked to survey responses. Completion of the survey implied consent.

### Statistical analysis

Data analysis was conducted using Stata version 17.1 (Stata Corp, College Station, TX). Descriptive statistics, including mean ± standard deviation (SD), median (range), or proportion (%), were predominantly employed to present results on participant demographics, model of care details, and clinicians’ perceptions, experience, and practices in measuring performance in the various domains as no hypothesis had been prespecified. Characteristic and response comparison were conducted between survey completers and non-completers, and between clinicians that had implemented professional role substitution models versus those that were in planning phases. The normality of data distribution was assessed using the Kolmogorov-Smirnov test and Q-Q plots. The Chi-square or Fisher’s Exact Test, and the Student’s t-test and Mann-Whitney U test were used for groups with normally and non-normally distributed data, respectively. In the question where participants were asked to allocate $100 across five strategies, a one-sample t-test was employed to compare mean allocations to the theoretical $20 for each strategy as per the null hypothesis. A p-value of < 0.05 indicated statistical significance. Content analysis and co-occurrence analysis were employed to summarise findings obtained from the open-ended questions in the survey.

## Results

### Study population

**Fig. 1:**
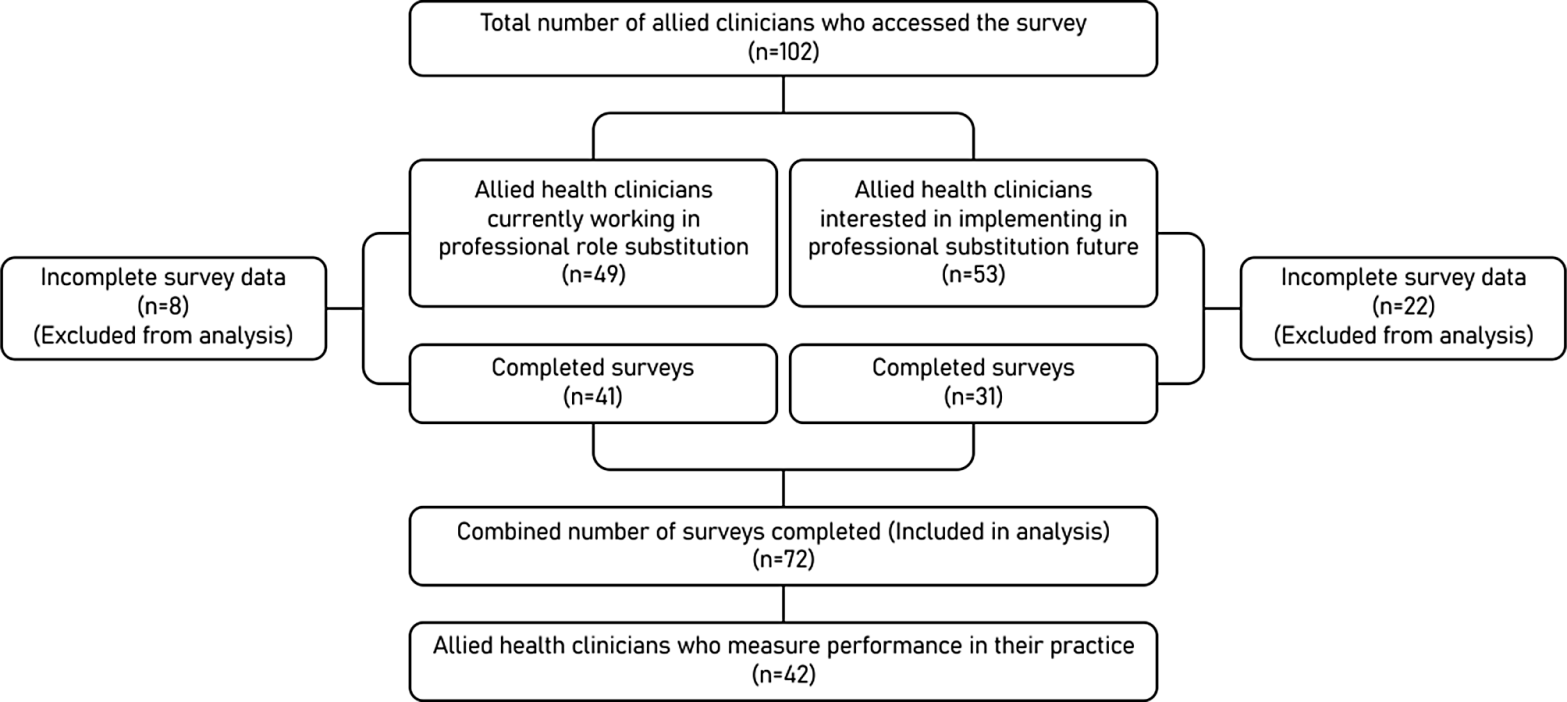
Allied clinicians professional role substitution survey respondents (*n* = 72)

Between June 22 and July 22, 2022, a total of 102 individuals accessed the survey, and 72 completed surveys were received (see Fig. 1). Forty-one surveys were completed by allied clinicians that were currently working in professional role substitution models of care and 31 were completed by clinicians in the planning stages of implementation. Due to the self-enrolment approach used in the study, it was not possible to estimate a response rate. There were no significant differences observed in terms of age (*p* = 0.08), allied health discipline (*p* = 0.263), years of experience as allied health professionals (*p* = 0.394), or education level (*p* = 0.699) between those who completed the survey and those who did not. However, individuals who completed the survey were more likely to be male (*p* = 0.009) and already involved in a professional role substitution model of care (*p* = 0.008).

### Representation

A broad representation from 13 out of 16 Queensland hospital and health services was achieved, with over two-thirds of responses originating from the State’s most populous hospital and health services, including Gold Coast (26.3%), Metro North (20.8%), and Metro South (19.4%). Table 1 provides an overview of the demographic characteristics of the respondents, who tended to be older and possess more professional experience compared to the average allied health workforce in Queensland [14]. 

**Table 1:** Participant characteristics

Demographic		Total (*n* = 72)	Currently involved in Professional Role Substitution(*n* = 41)	Interested in implementing Professional Role Substitution (*n* = 31)	P
**Age, years n (%)** ^**a**^					0.701
	18–25	2(2.8)	1(2.4)	1(3.2)	
	26–35	16(22.2)	8(19.5)	8(25.8)	
	36–45	32(44.4)	17(41.4)	15(48.4)	
	46–55	15(20.8)	11(26.8)	4(12.9)	
	56–65	7(9.7)	4(9.8)	3(9.7)	
**Gender n (%)** ^**b**^					0.922
	Male	19(26.4)	11(26.8)	8(25.8)	
	Female	53(73.6)	30(73.2)	23(74.2)	
**Allied-health discipline n (%)** ^**a**^					0.091
	Audiologist	5(6.9)	5(12.2)	0(0)	
	Dietitian/nutritionist	11(15.2)	8(19.5)	3(9.7)	
	Exercise physiologist	0(0)	0(0)	0(0)	
	Occupational therapist	9(12.5)	0(14.6)	5(16.1)	
	Optometrist	0(0)	0(0)	0(0)	
	Pharmacist	6(8.3)	1(2.4)	5(16.1)	
	Physiotherapist	16(22.2)	11(26.8)	5(16.1)	
	Podiatrist	6(8.33)	2(4.88)	4(12.9)	
	Psychologist	0(0)	0(0)	0(0)	
	Radiographer	1(1.4)	0(0)	1(3.23)	
	Social worker	2(2.78)	1(2.4)	1(3.2)	
	Speech pathologist	5(6.9)	3(7.3)	2(6.5)	
	Clinical measurements/cardiac or respiratory scientists	8(11.1)	2(4.9)	6(19.4)	
	Other	3(4.2)	2(4.9)	1(3.2)	
**Years as an allied-health professional, years n (%)** ^**a**^					0.183
	< 5	3(4.17)	0(0)	3(9.7)	
	6–10	12(16.7)	7(17.1)	5(16.3)	
	11–15	15(20.8)	9(22.0)	6(19.3)	
	16–25	28(38.9)	14(34.2)	14(45.2)	
	26–30	7(9.7)	6(14.6)	1(3.2)	
	> 30	7(9.7)	5(12.2)	2(6.4)	
**Education level n (%)** ^**a**^					0.198
	Bachelor’s degree	26(36.10	19(46.3)	7(22.6)	
	Graduate certificate	11(15.3)	5(12.2)	6(19.4)	
	Graduate diploma	7(9.7)	2(4.9)	5(16.1)	
	Master’s degree	25(34.7)	13(31.7)	12(38.7)	
	Doctoral degree	3(4.2)	2(4.9)	1(3.2)	
**Professional substitution role experience, years n (%)** ^**a**^					
	< 2		7(17.1)	Not applicable	
	2–5		12(29.3)		
	6–10		21(51.2)		
	> 10		1(2.4)		
Values are presented as proportions (%)					
^a^	Fisher’s exact test				
^b^	Chi-square test				

### Professional role substitution model of care details

A noticeable surge in interest in allied health professional role substitution models of care (MoC) over the past decade was evident, with clinicians in established professional role substitution models (*n* = 36; 87.8%) reporting the establishment of their MoC between 2011 and 2020. Professional role substitution MoC spanned across 12 specialties, with the highest responses coming from clinicians in otolaryngology (24.3%), gastroenterology (19.5%), orthopaedics (17.1%), and obstetrics and gynaecology (12.2%). The remaining responses were distributed across specialties including emergency medicine, endocrinology, mental health, neurosurgery, oncology and hematology, ophthalmology, respiratory and sleep medicine, and respiratory (26.9%). The majority primarily operated in specialist outpatient services (95%), with some also engaged in inpatient (22.0%), emergency department (10.0%), and subacute (7.3%) settings.

Activities undertaken by the 41 allied health clinicians working in professional role substitution models varied, with 51% involved in triaging activities, 85% conducting clinical histories, 68% performing physical examinations, 56% ordering tests, 22% reporting test results, 78% interpreting investigation results, 71% providing provisional diagnoses, 95% delivering patient management and treatment, 90% offering patient education, 22% prescribing or ordering medications, 73% making referrals to other health professionals, 78% discharging patients to GPs, and 20% performing procedures.

For those allied health clinicians yet to establish a professional role substitution model of care, there was notable interest in podiatry high-risk foot prescribing, pharmacist prescribing, and first-contact emergency department models of care. Survey participants were in the planning phase, with plans for implementation within the next 6–12 months.

### Allied-health clinicians’ perceptions, practice, and experiences of measuring performance

All respondents either “strongly agreed” (77.8%) or “agreed” (22.2%) that measuring the performance of MoCs was important. Clinicians deemed measuring performance crucial for improving patient outcomes and experiences (98.6%), enhancing efficiency and productivity (93.1%), evaluating cost-effectiveness (69.4%), supporting evidence-based policies (47.2%), identifying gaps and disparities (45.8%), and assisting patients in making informed healthcare decisions (38.9%). Measuring performance for regulatory compliance (15.3%) and recognising exceptional performance (6.9%) were considered less important.

### Allied-health clinicians’ perceptions on measuring various domains of healthcare quality

**Fig. 2:**
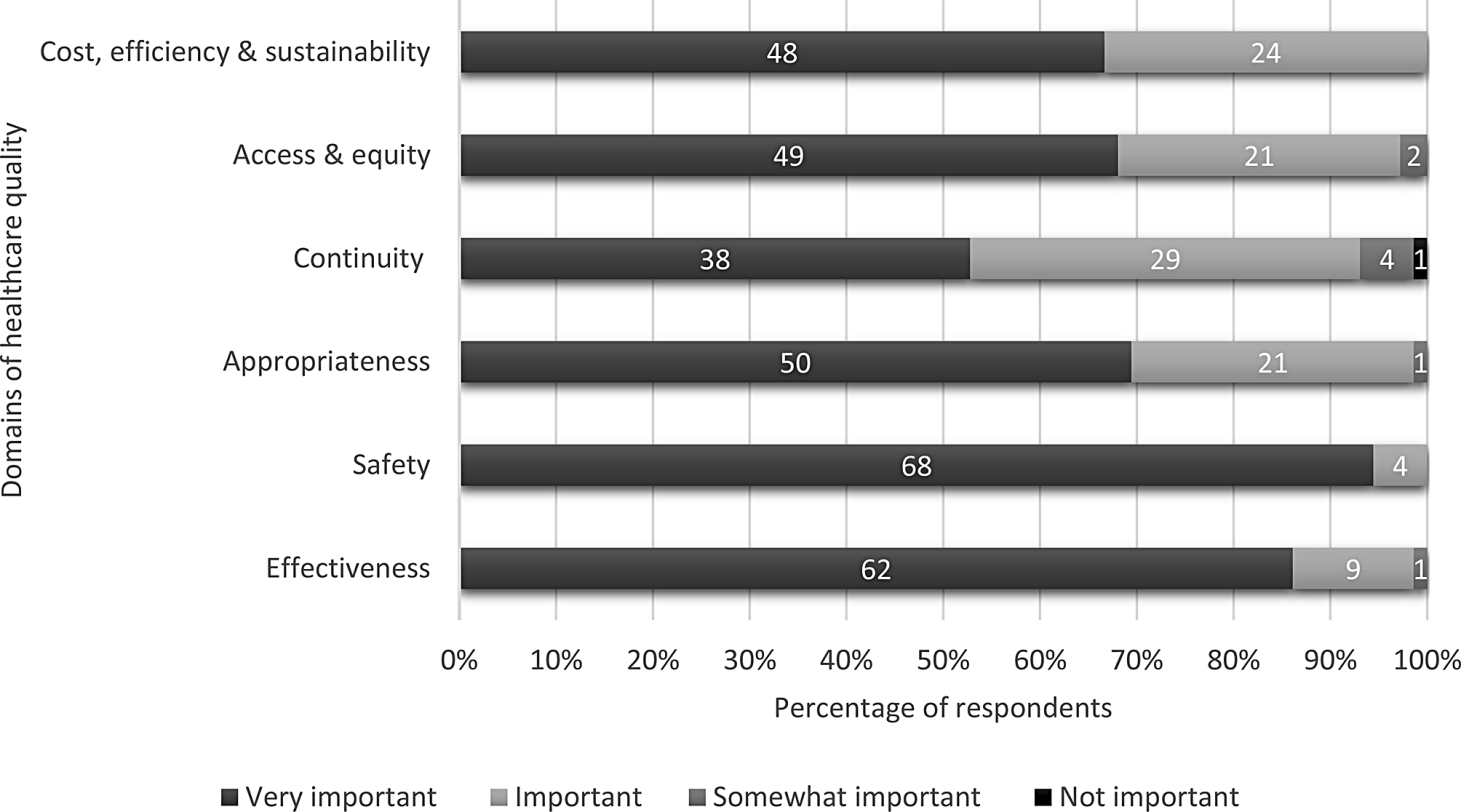
Allied-health clinicians’ perception of importance of measuring various domains of healthcare quality (*n* = 72)

Survey respondents were presented with six domains of healthcare quality (effectiveness, safety, appropriateness, access & equity, continuity of care, and cost, efficiency, productivity & sustainability) drawn from the National Academy of Medicine (NAM), formerly recognised as Institute of Medicine (IOM) healthcare quality dimensions [5], the Australian health performance framework [8], Levesque and Sutherland’s integrated performance evaluation framework [12], and a systematic review on measuring performance in professional role substitution models of care [7]. Most respondents believed that measuring six domains of healthcare quality was either “very important” or “important”. Safety (94.4%) and effectiveness (86.1%) were perceived as highly important, whereas 52.7% considered continuity and integration of care to be very important (Fig. 2).

### Measuring performance in practice

**Fig. 3:**
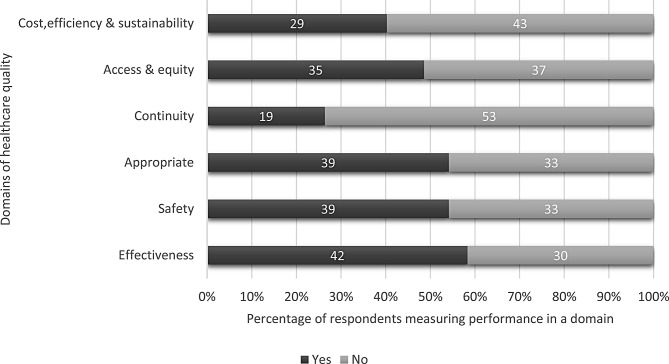
Allied-health clinicians’ current practice and patterns of measuring various domains of healthcare quality (*n* = 42)

While respondents expressed support for the principles of performance evaluation across healthcare quality domains, this support did not consistently translate into practice.58% of all respondents that completed the survey reported measuring the effectiveness of their MoCs. A smaller proportion (26%) measured continuity and integration of care. Allied-health clinicians actively working in role substitution MoCs were more likely to measure effectiveness (90.6% vs. 61.9%, *p* = 0.01) and cost (75.0% vs. 23.8%, *p* < 0.001) in their current roles compared to those who had not yet implemented a professional role substitution service. Only a third of those measuring performance reported using a framework, primarily relying on locally developed key performance indicators. Figure 3 provides an overview of the domains that were measured in practice, and Additional file 2 offers examples of measures used by clinicians.

### Enablers and barriers to measuring performance in professional role substitution MoC

**Fig. 4:**
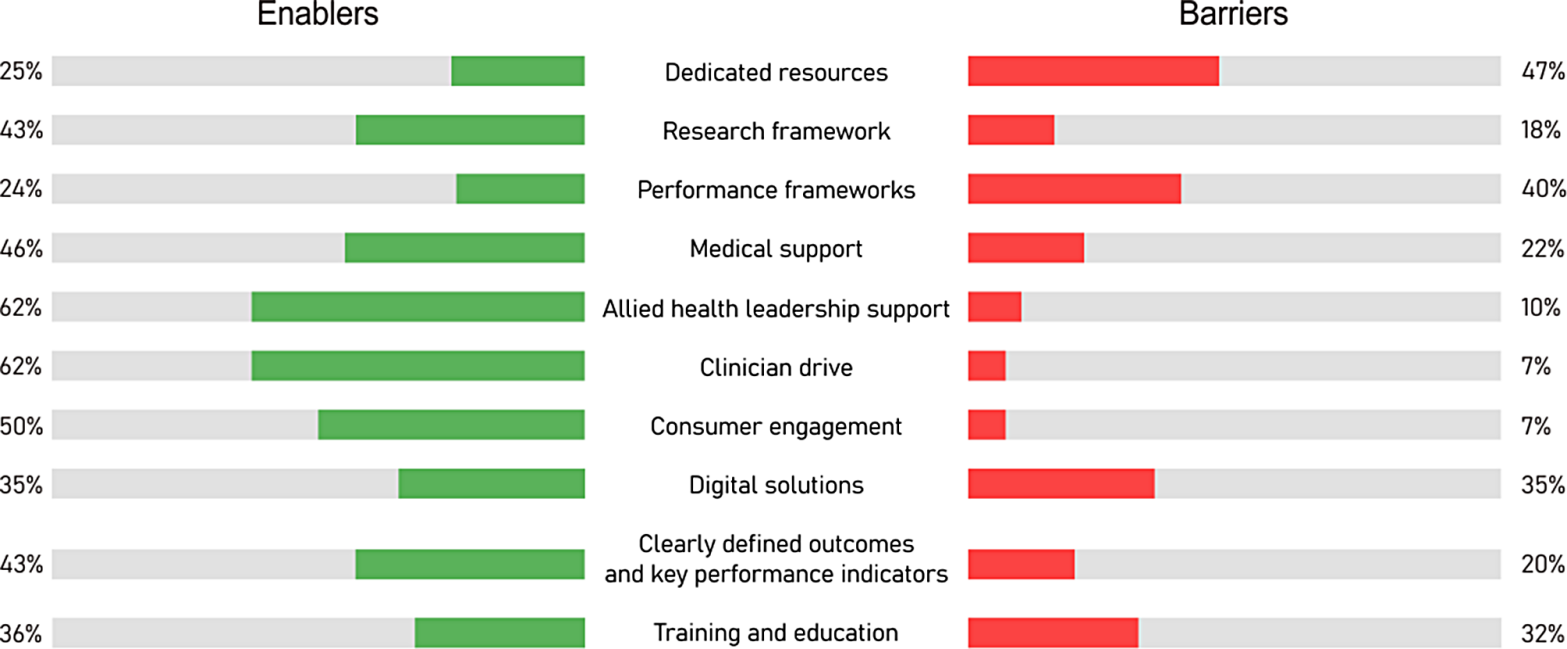
Proportion of allied-health clinicians reporting on various barriers and enablers to measuring performance in practice. (*n* = 72)

Enablers and barriers to performance evaluation in healthcare were identified among survey respondents. Figure 4 shows the proportion of respondents in the survey who identified the presented contextual factors as enablers or barriers in their practice. Key enablers included allied-health leadership support, identified by 62.5% of clinicians, clinician drive and motivation to promote performance evaluation (62.5%), consumer engagement (50%), and medical support (46%). Major barriers included a lack of resources (47%) and absence of healthcare performance frameworks and/or policies (40%). Allied-health clinicians involved in professional role substitution models of care were more likely to identify allied-health leadership (*p* = 0.04) and medical support (*p* = 0.001) as enablers compared to those yet to establish such models.

### What allied-health clinicians would be willing to spend on different strategies to support performance evaluation

Allied-health clinicians allocated significantly more financial resources to digital solutions facilitating systematic and standardised data collection, analysis, and reporting ($26.3, 95% CI [21.3, 31.3], *p* = 0.02) and the development of resource toolkits ($23.7, 95% CI [20.6, 27.0], *p* = 0.02). In contrast, less investment was directed towards financial incentives ($4.81, 95% CI [3.1, 6.6], *p* < 0.001). The mean allocated funding for the development of frameworks ($21.9, 95% CI [17.9, 25.8], *p* = 0.33) and for education and training ($23.0, 95% CI [18.7–27.5], *p* = 0.16) did not significantly differ from the expected equal distribution of funding. These results were consistent regardless of whether participants were already working in professional role substitution or planning to establish such models.

### Dissemination strategies for performance evaluation findings

**Fig. 5:**
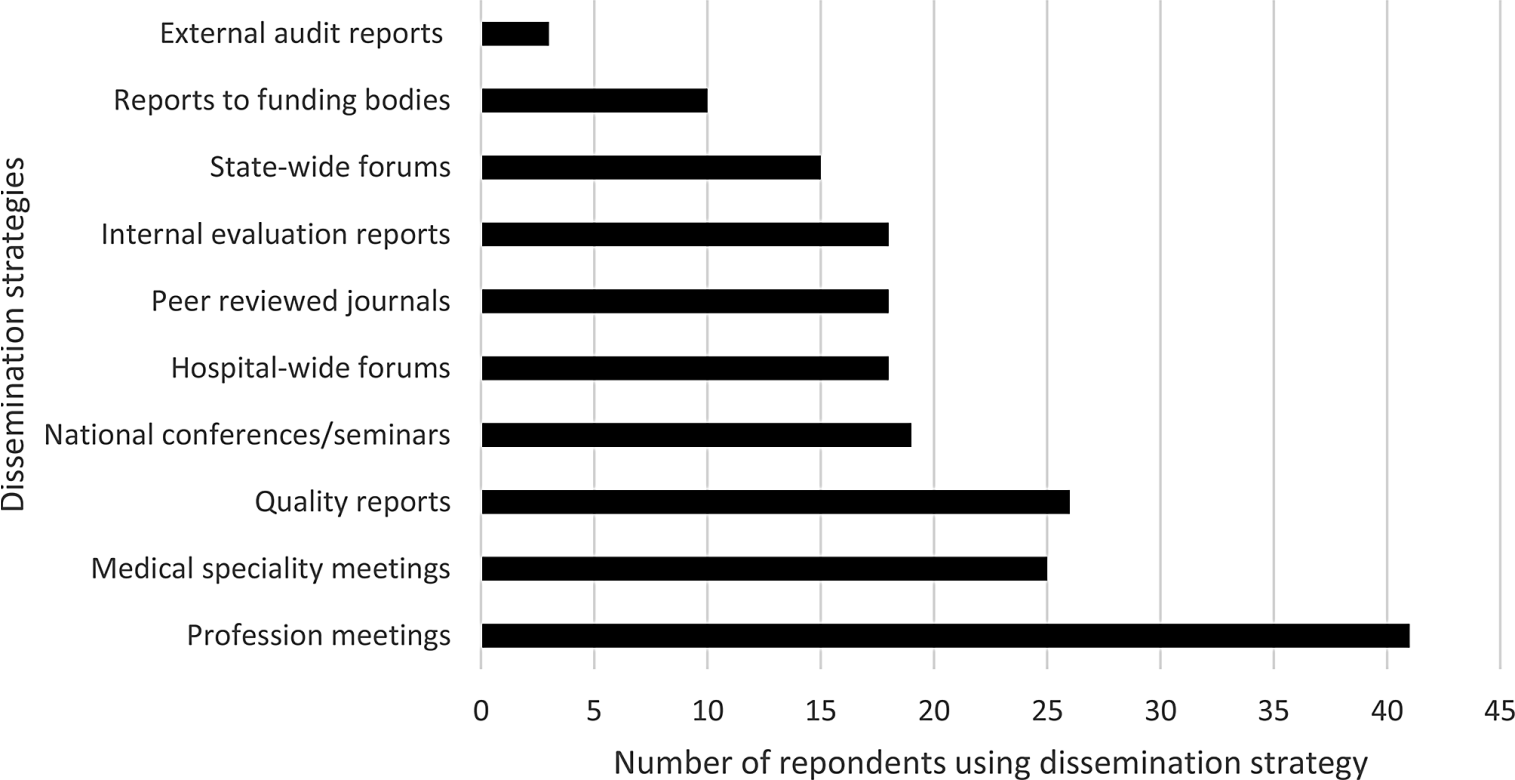
Dissemination strategies used by allied-health clinicians to report performance evaluation (*n* = 42)

Among the allied-health clinicians who measured performance various strategies were employed to report and disseminate findings of performance evaluation (Fig. 5). The most common approach was presenting findings at local professional meetings (e.g., health service physiotherapy department meetings). Notably, allied health clinicians engaged in professional role substitution models of care were more likely to present their findings at specialty meetings (*p* = 0.02), State-wide forums (*p* = 0.001), conferences/seminars (*p* = 0.005), and in peer-reviewed journals (*p* = 0.002).

## Discussion

This study reveals an increase in the adoption of allied-health professional role substitution models of care (MoC) over the past decade, with implementation across a wide range of medical specialties. However, despite this growth, the translation of principles supporting healthcare performance evaluation into everyday practice remains limited. Only half of the surveyed clinicians reported measuring one or more domains of health quality. This finding aligns with our recent systematic review, which underscores the substantial gaps in performance evaluation of non-medical professional role substitution MoCs [7]. Although there is near-universal consensus on the potential benefits of performance evaluation in healthcare, there is limited evidence to guide this practice, and issues concerning strategic alignment and criteria for selecting measures continue to be subjects of debate [21]. Inadequate performance evaluation may impact adoption and scalability of models of care. Where models of care are not effective there is a risk of continued provision of suboptimal quality care with potential impact on patient outcomes.

Previously identified challenges that can stifle implementation of performance evaluation warrant consideration. Time, manpower and cost associated with the process of undertaking performance evaluation are often reported as significant barriers, being limited within the constraints of healthcare systems [22–24]. Our study echoes this, with clinicians indicating a lack of resources as an ongoing barrier to performance evaluation. However, allied-health leadership, medical leadership and support, and clinician drive were identified as important enablers. This likely reflects the broad stakeholder engagement associated with the implementation of the allied health expanded scope of practice strategy in Queensland Health [1, 15, 16]. 

There is growing advocacy for active patient involvement in the care delivery process, as this has been associated with improved healthcare quality, outcomes, and cost containment [25]. Importantly, our study underscores the often-overlooked importance of consumer engagement in performance evaluation. This highlights the need to move beyond mere rhetoric concerning patient engagement during the planning and implementation of initiatives. Methods to encourage and monitor patient engagement when measuring healthcare performance should also be considered. Overall strategies may include patient education, shared decision making, encouraging patients to take initiative to learn and take ownership of their care and encouraging patients to actively engage in healthcare improvement strategies, of which performance evaluation is a key component [25]. 

Clinicians indicated that satisfying regulatory requirements or providing financial rewards for performance were less important aspects of measuring performance. Consistent with this, clinicians allocated the least financial resources to incentivising strategies to improve performance evaluation. There is evidence to suggest that financial rewards have mixed effects on performance, with some studies indicating that recognition is a more important reward for healthcare professionals [26]. This suggests that healthcare leaders and policy makers need to focus on other strategies to encourage performance evaluation in the models.

Notably, investment in digital platforms emerged as the preferred clinician-identified strategy to support and promote performance evaluation in professional role substitution MoC. Given the opportunity, clinicians would allocate more financial resources to this. Information systems have the potential to provide essential data that can be used in healthcare performance assessment and management. However, assessment is likely to be opportunistic, reflecting availability of data rather than alignment with organisational strategic goals [27]. This makes it difficult to determine the true value of new healthcare delivery models, including allied-health professional role substitution MoC. Technology can be used to automate and streamline data collection with potential to provide real-time feedback. This can lead to a more efficient and transparent performance evaluation process that benefits patients, clinicians and the healthcare system [27]. 

While there is consensus on the need to measure, analyse, and disseminate healthcare performance findings, there is little agreement on how data should be reported and whether different formats are required for different audiences [27]. Healthcare is rich in evidence-based innovations, including allied-health professional role substitution MoCs [28]. However, even when such innovations are successfully implemented at one site, their uptake at other locations is often slow, if it occurs at all [7]. This study found that evaluations of allied-health professional role substitution MoCs were primarily disseminated at a local level. Innovative MoCs that do not effectively report on performance may have limited long-term impact. Localised reporting, rather than wider publication, may be overlooked in literature reviews and evidence syntheses often used to inform policy and practice [29]. There maybe benefit integrating research frameworks when implementing new MoC, which clinicians in this study also highlighted as an enabler to performance evaluation. This study highlighted very limited use of external audits (review that is conducted by a party not associated with the company or department) to evaluate performance of professional role substitution MoC. This is an area that warrants further attention with potential to improve performance evaluation, reduce bias, as well as build on the quality and validity of evaluation efforts.

### Strengths and limitations

This study is the first to our knowledge to explore allied-health clinicians’ practice, perceptions, and experiences with measuring performance of professional role substitution MoC in any location. We have identified barriers and enablers to inform strategies to help clinicians in these roles improve performance evaluation. This is integral to in-service transformation and ensures the highest quality of patient care through transparency, accountability, and credibility [30]. Whilst it was not possible to determine the response rate, there was a high level of engagement of the study population with 72 respondents across approximately 133 distinct models reported in the State. A further strength is that the responding cohort were derived from a range of hospital and health services across a large state in Australia, from both tertiary and regional/rural settings, and representing clinicians from a range of disciplines and medical specialities.

As with most surveys, respondents with biases may have self-selected into the sample. The survey population was restricted to clinicians working on or intending to implement professional role substitution MoCs within an Australian state with an established expanded scope of practice strategy. Thus, the generalisability of survey results to other states or countries should be considered when interpreting the results. Future studies could extend survey to a wider range of settings. In measuring performance in healthcare, the perspectives of various stakeholders are valuable. Thus, future research should explore the perspectives of other stakeholders, including managers, health executives, medical practitioners, and policymakers.

## Conclusions

In conclusion, allied-health professionals working in or interested in professional role substitution MoCs express support for principles of performance evaluation. However, this support does not consistently translate into practice, with inadequate performance evaluation observed across the various domains which reflect healthcare quality. Organisational strategies can maximise enablers while addressing the identified barriers to improve performance evaluation in these models of care.

### Electronic supplementary material

Below is the link to the electronic supplementary material.

**Supplementary Material 1**: Measuring performance in allied health professional role substitution models of care- clinician survey

**Supplementary Material 2**: Summary of outcomes measured by clinicians across six domains of quality

## Data Availability

Data are available from corresponding author on request.

## Abbreviations

VBHC: Value-Based Healthcare
AMEE: Association of Medical Education in Europe
CVR: Content Validity Ratios
AHPOQ: Allied Health Professions Queensland
MoC: Models of Care
IOM: Institute of Medicine Dimensions
