# Supplementary material for: Measuring performance in allied health professional role substitution models of care: a clinician survey
Source: BMC Health Serv Res. 2024 Jan 16;24:79. doi: 10.1186/s12913-024-10556-5 (PMC10790527; doi:10.1186/s12913-024-10556-5)
Supplement: Supplementary file 1 — Supplementary Material 1: Measuring performance in allied health professional role substitution models of care- clinician survey [file 12913_2024_10556_MOESM1_ESM.pdf]

Measuring Performance in Allied Health Professional Role Substitution Models of Care- Clinician Survey

Thank you for your interest in this study about measuring performance in professional role substitition models of care.

You can read more about the study in the Participant Information and Consent Statement.

The survey is being conducted using RedCap which is based in the Australia. Information you provide on this survey will be transferred using this platform.

By completing this survey, you agree to this transfer of information, indicate that you understand the information supplied and you consent to taking part in the study.

Click "next page" to complete the survey.

[Attachment: "Participant Information and Consent Form (PICF).pdf"]

Part A : Participant demographics

What is your age group?

- ◯ 18-25 years
- ◯ 26-35 years
- ◯ 36-45 years
- ◯ 46-55 years
- ◯ 56-65 years
- ◯ Over 65 years

How do you describe your gender?

- ◯ Male
- ◯ Female
- ◯ Non-Binary
- ◯ Another term (please specify)
- ◯ Prefer not to say

Please specify

____

What is your allied health discipline?

- ◯ Audiologist
- ◯ Dietitian or nutritionist
- ◯ Exercise physiologist
- ◯ Occupational therapist
- ◯ Optometrist
- ◯ Pharmacist
- ◯ Physiotherapist
- ◯ Podiatrist
- ◯ Psychologist
- ◯ Radiographer /medical imaging technologists
- ◯ Social worker
- ◯ Speech pathologist
- ◯ Other (please specify)

Please specify

____

How long have you worked as an allied health professional?	◯ < 5 years<br>◯ 6-10 years<br>◯ 11-15 years<br>◯ 16-25 years<br>◯ 26-30 years<br>◯ >30 years
What is your highest level of education?	◯ Bachelor's degree<br>◯ Graduate certificate<br>◯ Graduate diploma<br>◯ Master's degree<br>◯ Doctoral degree<br>◯ Other (please specify)
Please specify	__________________________________

Participant workplace details

Which hospital and health service do you predominantly work in Queensland?	◯ Cairns and Hinterland<br>◯ Central Queensland<br>◯ Central West<br>◯ Children's Health Queensland<br>◯ Darling Downs<br>◯ Gold Coast<br>◯ Mackay<br>◯ Metro North<br>◯ Metro South<br>◯ North West<br>◯ South West<br>◯ Sunshine Coast<br>◯ Torres and Cape<br>◯ Townsville<br>◯ West Moreton<br>◯ Wide Bay<br>◯ Other
Do you currently work or have you previously worked in a professional role substitution model of care?	◯ Yes<br>◯ No
How long have you worked in a professional substitution role?	◯ < 2 years<br>◯ 2-5 years<br>◯ 5-10 years<br>◯ >10 years

Part B: Profession role substitution model of care details

What is the name of your professional role substitution model of care?<br><br>(e.g. Physiotherapy vestibular primary contact clinic, Speech pathology-led ENT service, Dietitian first gastroenterology clinic, Pharmacist prescibing,)	__________________________________
What professional role substitution model are you interested in implementing?	__________________________________
What year was the professional role substitution model of care implemented in your service?	__________________________________
When are you planning to start the professional role substitution model of care?	◯ < 6 months<br>◯ 6-12 months<br>◯ 13-24 months<br>◯ > 2 years
Which medical speciality is the professional substitution role aligned with?	◯ Cardiology<br>◯ Emergency medicine<br>◯ Endocrinology<br>◯ Gastroenterology and hepatology<br>◯ General medicine<br>◯ General surgery<br>◯ Mental health<br>◯ Nephrology<br>◯ Neurology<br>◯ Neurosurgery<br>◯ Obstetrics and Gynaecology<br>◯ Oncology and hematology<br>◯ Ophthalmology<br>◯ Orthopaedics<br>◯ Otolaryngology (ear, nose, throat, head, and neck)<br>◯ Paediatrics<br>◯ Persistent pain management<br>◯ Plastic and reconstructive surgery<br>◯ Respiratory and sleep medicine<br>◯ Rheumatology<br>◯ Urology<br>◯ Vascular<br>◯ Other(Please specify)

Which medical speciality would the professional substitution role be aligned with?	◯ Cardiology<br>◯ Emergency medicine<br>◯ Endocrinology<br>◯ Gastroenterology and hepatology<br>◯ General medicine<br>◯ General surgery<br>◯ Mental health<br>◯ Nephrology<br>◯ Neurology<br>◯ Neurosurgery<br>◯ Obstetrics and Gynaecology<br>◯ Oncology and hematology<br>◯ Ophthalmology<br>◯ Orthopaedics<br>◯ Otolaryngology (ear, nose, throat, head, and neck)<br>◯ Paediatrics<br>◯ Persistent pain management<br>◯ Plastic and reconstructive surgery<br>◯ Respiratory and sleep medicine<br>◯ Rheumatology<br>◯ Urology<br>◯ Vascular<br>◯ Other(Please specify)
Please specify	__________________________________
What clinical setting do you work in?(Tick all that apply)	☐ Outpatients<br>☐ Inpatients<br>☐ Emergency department<br>☐ Sub-acute<br>☐ Other(please specify)
Which clinical setting would the professional substitution role work in? (Tick all that apply)	☐ Outpatients<br>☐ Inpatients<br>☐ Emergency department<br>☐ Sub-acute and ambulatory care<br>☐ Other(please specify)
Please specify	__________________________________
What activities do you perform as part of your role in the professional role substitution model of care? (Please tick all that apply)	☐ Triage<br>☐ Taking a clinical history<br>☐ Performing physical examinations<br>☐ Ordering tests (e.g., blood tests , x-rays)<br>☐ Reporting tests (e.g., radiology images)<br>☐ Interpreting results of investigations<br>☐ Providing provisional diagnosis<br>☐ Patient management and treatment<br>☐ Patient education<br>☐ Prescribing or ordering medications<br>☐ Referrals to other health professionals<br>☐ Discharge patients to general practitioners(GP)<br>☐ Performing procedures (e.g., inserting nasogastric or tracheostomy tubes, injecting, or suturing)<br>☐ Other (Please specify)

What activities will/would you perform as part of your role in the professional role substitution model of care? (Please tick all that apply)	☐ Triage<br>☐ Taking a clinical history<br>☐ Performing physical examinations<br>☐ Ordering tests (e.g., blood tests , x-rays)<br>☐ Reporting tests (e.g., radiology images)<br>☐ Interpreting results of investigations<br>☐ Providing provisional diagnosis<br>☐ Patient management and treatment<br>☐ Patient education<br>☐ Prescribing or ordering medications<br>☐ Referrals to other health professionals<br>☐ Discharge patients to general practitioners(GP)<br>☐ Performing procedures (e.g., inserting nasogastric or tracheostomy tubes, injecting, or suturing)<br>☐ Other (Please specify)
Please specify	__________________________________

Part C: Measuring Performance of Professional Role Substitution Models of Care- Clinician Attitudes

To what extent do you agree with the following statement?

Measuring or evaluating the performance of a model of care is important.

◯ Strongly agree
◯ Agree
◯ Somewhat agree
◯ Somewhat disagree
◯ Disagree
◯ Strongly disagree

Measuring the performance of professional role substitution models of care is important because it can...
(Tick up to 5 of the most important to you)

☐ Improve patient outcomes and care experiences
☐ Evaluate cost of service delivery
☐ Identify gaps and disparities in healthcare
☐ Improve efficiency and productivity in healthcare delivery
☐ Satisfy the regulatory requirements
☐ Ensure adherence to standards or benchmarks
☐ Support patients in making informed healthcare decisions
☐ Support governments, policy makers and health services to make evidence-based policies and investments
☐ Recognise and reward above-standard performance
☐ Secure ongoing data to monitor changes in quality of care over time
☐ Other (please specify)

CHECKED BOXES IS:

____

Please specify

____

The domains below have been used previously to categorise performance measures. Please read the information provided in the table to help you answer the questions that follow.
Domain

Definition

Examples of indicators

1) Effectiveness

Care, intervention, or action achieves desired outcomes

o Generic and disease specific outcomes (e.g., short form 36, RAQoL)

o Re-admissions, revisions, re-referrals

2) Safety

The avoidance or reduction of actual or potential harm from healthcare delivery

o Diagnostic accuracy, missed diagnosis

o Guideline/protocol concordant care

3) Appropriateness and responsiveness

Service is person centred and culturally appropriate

o Patient experience and satisfaction

o Referrer satisfaction

4) Continuity of care and integration

Ability to provide uninterrupted care

o Rates of appropriate follow up

o Clinician or management continuity

5) Accessibility and equity

Timely access, with skills and resources appropriate to medical need

- **Access metrics (e.g., wait-time)**
- **Healthcare utilisation measures**

6) Cost, efficiency, productivity, and sustainability

Delivering care in a manner which maximises resources use and avoids waste

- **Cost-effectiveness or benefit**
- **Measures of willingness to pay**

How important is it to measure the following domains in professional role substitution models of care?

	Very important	Important	Somewhat important	Not important
1) Effectiveness	○	○	○	○
2) Safety	○	○	○	○
3) Appropriateness and responsiveness	○	○	○	○
4) Continuity of care and integration	○	○	○	○
5) Accessibility and equity	○	○	○	○
6) Cost , efficiency, productivity, and sustainability	○	○	○	○

Part D: Measuring performance in practice

With the professional role substitution model of care in mind,

Do you measure performance in this service?

◯ Yes ◯ No

Do you currently measure performance in your role?

◯ Yes ◯ No

Do you use a framework to measure performance in your service?

◯ Yes ◯ No

What is the name of the framework?

____

Here again is the table of domains previously used to measure performance. Please use this as a reference to help you answer the following question. Domain

Definition

Examples of indicators

1) Effectiveness

Care, intervention, or action achieves desired outcomes

o Generic and disease specific outcomes (e.g., short form 36, RAQoL)

o Re-admissions, revisions, re-referrals

2) Safety

The avoidance or reduction of actual or potential harm from healthcare delivery

o Diagnostic accuracy, missed diagnosis

o Guideline/protocol concordant care

3) Appropriateness and responsiveness

Service is person centred and culturally appropriate

o Patient experience and satisfaction

o Referrer satisfaction

4) Continuity of care and integration

Ability to provide uninterrupted care

o Rates of appropriate follow up

o Clinician or management continuity

5) Accessibility and equity

Timely access, with skills and resources appropriate to medical need

- **Access metrics (e.g., wait-time)**
- **Healthcare utilisation measures**

6) Cost, efficiency, productivity, and sustainability

Delivering care in a manner which maximises resources use and avoids waste

- **Cost-effectiveness or benefit**
- **Measures of willingness to pay**

What domains do you measure in your service?

	Yes	No
1) Effectiveness	◯	◯
Examples of the indicators you use to measure effectiveness	________________	
2) Safety	◯	◯
Examples of the indicators you use to measure safety	________________	
3) Appropriateness and responsiveness	◯	◯
Examples of the indicators you use to measure appropriateness and responsiveness	________________	
4) Continuity of care and integration.	◯	◯
Examples of the indicators you use to measure continuity of care and integration	________________	

5) Accessibility and equity	Yes ○	No ○

Examples of the indicators you use to measure access and equity

____

6) Cost efficiency, productivity, and sustainability	Yes ○	No ○

Examples of the indicators you use to measure cost efficiency , productivity and sustainability

____

7) Other (Please provide details of any other the indicators you measure)

____

How do you disseminate your findings? (Please tick all that apply)

- ☐ Local profession meetings (e.g. physiotherapy department meetings)
- ☐ Local specialty meetings (e.g. gastroenterology department meetings)
- ☐ Quality improvement reports
- ☐ Hospital and health service- wide platforms
- ☐ State-wide forums
- ☐ Conferences /seminars
- ☐ Peer reviewed publications
- ☐ Reports to funding bodies
- ☐ Internal evaluations
- ☐ External audits
- ☐ Other (please specify)

Please specify

____

Part E: Barriers and enablers to measuring performance

Previously, the availability or lack of the following elements have been found to impact performance measurement in healthcare.

Please indicate whether each element has been an enabler (available) or barrier (lacks) in your service.

		Enabler	Barrier	Not applicable
a)	Dedicated resources (e.g. financial, human, time)	○	○	○
b)	Integration of performance measurement in research or evaluation projects	○	○	○
c)	Healthcare performance frameworks and/or policies	○	○	○
d)	Medical leadership and support	○	○	○
e)	Allied health leadership and support	○	○	○
f)	Clinician drive	○	○	○
g)	Consumer support and engagement	○	○	○
h)	Digital solutions (e.g., data management systems)	○	○	○
i)	Clear and well defined performance indicators and outcome measures	○	○	○
j)	Training and education to support measuring performance in healthcare	○	○	○
k)	Collaboration across sites	○	○	○

Other enablers (please specify) __________________________________

Other barriers (please specify) __________________________________

Part F: Measuring performance in professional role substitution models of care - Future Strategies

If you were given a total of $100 to spend on strategies to support measuring performance in professional role substitution models of care, how much would you allocate to each of these five (5) areas?

a) Development of frameworks with clear principles and processes

$0 $50 $100

(Place a mark on the scale above)

b) A tool kit with well developed resources

$0 $50 $100

(Place a mark on the scale above)

c) Digital solutions that facilitate systematic and standardised data collection, analysis and reporting.

$0 $50 $100

(Place a mark on the scale above)

d) Financial incentives

$0 $50 $100

(Place a mark on the scale above)

e) Education and training programs

$0 $50 $100

(Place a mark on the scale above)

(Check total is: $100) ______________________

Your current total is: $[strategies_total]

**The total is less than $100

**The total is more than $100

√ The total is equal to $100

Do have any other comments before you finish this survey? ______________________

Part G : Participation in future research on this topic and draw

Please note: If you choose to provide contact details, they will not be linked to your survey data, your anonymity will be maintained in reporting and your contact details will only be used by the research team for the purposes of inviting you to participate in future research or the draw. You may decline further participation at any time.

There is an opportunity to provide further feedback to the researchers on this topic. Do you consent to be contacted to participate in face to face or telephone interviews?	◯ Yes<br>◯ No
Do you want to enter draw for your chance to win one of three (3) $100 Coles-Myer gift cards?	◯ Yes<br>◯ No
Name	__________________________
Email	__________________________
Phone Number	__________________________
