# Supplementary material for: Measuring performance in allied health professional role substitution models of care: a clinician survey
Source: BMC Health Serv Res. 2024 Jan 16;24:79. doi: 10.1186/s12913-024-10556-5 (PMC10790527; doi:10.1186/s12913-024-10556-5)
Supplement: Supplementary file 2 — Supplementary Material 2: Summary of outcomes measured by clinicians across six domains of quality [file 12913_2024_10556_MOESM2_ESM.docx]

| **Supplementary Table 1.** Summary of outcomes measured by clinicians across six domains of quality | | | | | |
| --- | --- | --- | --- | --- | --- |
| **Effectiveness** | **Safety** | **Appropriateness** | **Continuity of Care and Integration** | **Accessibility** | **Efficiency (incl economic analysis)** |
| - Medical speciality visits - Overall - Primary presenting reason - Discharge rates Hospitalisations - Overall - Primary presenting reason - Re-referral rates - Recovery rates - Resolution of concerns (e.g ulcer healing rate) - Symptom, pain or function (e.g., global rating of change, IBS symptom severity) - Health status and quality of life (IBS Quality of life scale, Euro QoL 5D) - Patient adherence - Patient enablement index/score (extent to which patients understand their illness and are able to cope) - Patient compliance scores | - Missed or delayed diagnosis rates - Inter rater reliability Surgical conversion rates - Complication rates or adverse events - Error rates - Diagnostic accuracy and yield - Diagnostic accuracy: Sensitivity and specificity - Misdiagnosis rates - Complaints | - Patient satisfaction measured by; - Satisfaction surveys - Patient feedback - Review of complaints and compliments - Stakeholder surveys - Patients experience as part research projects | - Number of follow up appointments - Appropriate follow up rates by medical specialist | - Healthcare utilisation - Wait time - Time to discharge - Time from referral to procedure - Time to consultation - Service uptake - Proportion of patients in breach of recommended wait times - Length of stay or episode of care. | - Cost of avoiding investigations and procedures - Inputs and health resources utilisation (e.g QWAUS generated versus cost) - Cost effectiveness studies in conjunction with a heath economist - Cost of implementation (labour and non-labour) compared to revenue (QWAU or $) |
